# Safety and Efficacy of the Intravenous Infusion of Umbilical Cord Mesenchymal Stem Cells in Patients With Heart Failure: A Phase 1/2 Randomized Controlled Trial (RIMECARD Trial [Randomized Clinical Trial of Intravenous Infusion Umbilical Cord Mesenchymal Stem Cells on Cardiopathy])

**DOI:** 10.1161/CIRCRESAHA.117.310712

**Published:** 2017-10-26

**Authors:** Jorge Bartolucci, Fernando J. Verdugo, Paz L. González, Ricardo E. Larrea, Ema Abarzua, Carlos Goset, Pamela Rojo, Ivan Palma, Ruben Lamich, Pablo A. Pedreros, Gloria Valdivia, Valentina M. Lopez, Carolina Nazzal, Francisca Alcayaga-Miranda, Jimena Cuenca, Matthew J. Brobeck, Amit N. Patel, Fernando E. Figueroa, Maroun Khoury

**Affiliations:** From the Laboratory of Nano-Regenerative Medicine (J.B., P.L.G., F.A.-M., J.C., F.E.F., M.K.) and Department of Internal Medicine (F.J.V., R.E.L., F.E.F.), Faculty of Medicine, Universidad de los Andes, Santiago, Chile; Department of Cardiology, Clínica Santa Maria, Santiago, Chile (J.B., E.A., C.G., R.L., P.A.P., G.V.); Program for Translational Research in Cell Therapy, Clínica Universidad de los Andes, Santiago, Chile (J.B., F.J.V., F.E.F., M.K.); Consorcio Regenero, Chilean Consortium for Regenerative Medicine, Santiago, Chile (P.L.G., F.A., J.C., F.E.F., M.K.); Department of Cardiology, Clínica Davila, Santiago, Chile (R.E.L., P.R., I.P.); Cells for Cells, Santiago, Chile (V.M.L., M.K.); Public Health School, Faculty of Medicine, Universidad de Chile, Santiago, Chile (C.N.); Division of Physical Medicine Rehabilitation, University of Utah, Salt Lake City (M.J.B.); and Department of Surgery, University of Miami School of Medicine, FL (A.N.P.).

**Keywords:** cardiomyopathies, clinical trial, heart failure, mesenchymal stromal cells, umbilical cord

## Abstract

**Rationale:**

Umbilical cord–derived mesenchymal stem cells (UC-MSC) are easily accessible and expanded in vitro, possess distinct properties, and improve myocardial remodeling and function in experimental models of cardiovascular disease. Although bone marrow–derived mesenchymal stem cells have been previously assessed for their therapeutic potential in individuals with heart failure and reduced ejection fraction, no clinical trial has evaluated intravenous infusion of UC-MSCs in these patients.

**Objective:**

Evaluate the safety and efficacy of the intravenous infusion of UC-MSC in patients with chronic stable heart failure and reduced ejection fraction.

**Methods and Results:**

Patients with heart failure and reduced ejection fraction under optimal medical treatment were randomized to intravenous infusion of allogenic UC-MSCs (Cellistem, Cells for Cells S.A., Santiago, Chile; 1×10^6^ cells/kg) or placebo (n=15 per group). UC-MSCs in vitro, compared with bone marrow–derived mesenchymal stem cells, displayed a 55-fold increase in the expression of hepatocyte growth factor, known to be involved in myogenesis, cell migration, and immunoregulation. UC-MSC–treated patients presented no adverse events related to the cell infusion, and none of the patients tested at 0, 15, and 90 days presented alloantibodies to the UC-MSCs (n=7). Only the UC-MSC–treated group exhibited significant improvements in left ventricular ejection fraction at 3, 6, and 12 months of follow-up assessed both through transthoracic echocardiography (*P*=0.0167 versus baseline) and cardiac MRI (*P*=0.025 versus baseline). Echocardiographic left ventricular ejection fraction change from baseline to month 12 differed significantly between groups (+7.07±6.22% versus +1.85±5.60%; *P*=0.028). In addition, at all follow-up time points, UC-MSC–treated patients displayed improvements of New York Heart Association functional class (*P*=0.0167 versus baseline) and Minnesota Living with Heart Failure Questionnaire (*P*<0.05 versus baseline). At study completion, groups did not differ in mortality, heart failure admissions, arrhythmias, or incident malignancy.

**Conclusions:**

Intravenous infusion of UC-MSC was safe in this group of patients with stable heart failure and reduced ejection fraction under optimal medical treatment. Improvements in left ventricular function, functional status, and quality of life were observed in patients treated with UC-MSCs.

**Clinical Trial Registration:**

URL: https://www.clinicaltrials.gov/ct2/show/NCT01739777. Unique identifier: NCT01739777

## Abstract 2

Supplemental Digital Content is available in the text.

Stem cell therapy has been under evaluation as a treatment for heart failure (HF) with reduced ejection fraction (HFrEF) for more than a decade. Experimental studies report improvements in cardiac function and regeneration of damaged heart tissue through mechanisms, including transdifferentiation, cell fusion, and paracrine modulation.^1,2^ In human disease, recent reviews suggest that stem cell therapy is safe and associated with moderate clinical benefits in survival, left ventricular function, and quality of life of patients with HFrEF.^3–6^ Clinical trials in patients with chronic ischemic or nonischemic disease have assessed a range of cellular products and delivery routes. These include autologous or allogenic bone marrow mononuclear cells and mesenchymal stem cells (MSC), administered by intramyocardial injections, percutaneous intracoronary infusion, and exceptionally peripheral intravenous infusion.^3,4,6^ However, after decades of basic and clinical research, overall benefit and the best cell source and route of administration remain unsettled.

**Editorial, see p 1116**

**In This Issue, see p 1103**

**Meet the First Author, see p 1104**

MSCs are multipotent cells with low immunogenic potential that can be isolated from adult tissues, including bone marrow, adipose tissue, and umbilical cord among other sources. The niche of origin represents an essential factor when evaluating biological differences between cell types because MSC properties can be highly influenced by microenvironmental changes.^1,7^ Most experimental and clinical studies have used bone marrow–derived MSC (BM-MSC), nonetheless these cells present disadvantages for clinical application, including an invasive harvesting procedure and a decreased proliferation and differentiation potential related to donor age and comorbidity.^8^ In contrast, umbilical cord-derived MSCs (UC-MSC) are easily attainable and expanded in vitro, have less cellular aging, and are devoid of ethical concerns. Preclinical studies have demonstrated that UC-MSC can express cardiac-specific molecules (troponin-I, connexin-43), differentiate into cardiomyocyte-like and endothelial cells in vitro, and also exert paracrine effects that enhance vascular regeneration and cardiomyocyte protection. Such actions might underlie the improvement in cardiac function observed in animal models of chronic ischemic cardiomyopathy and dilated cardiomyopathy in response to UC-MSCs.^9–14^ The aim of this prospective, randomized, double blinded placebo–controlled trial was to evaluate the safety and efficacy of a well-characterized source of UC-MSCs administered intravenously in patients with chronic HFrEF.

## Methods

### Study Design and Patient Population

The RIMECARD trial (Randomized Clinical Trial of Intravenous Infusion Umbilical Cord Mesenchymal Stem Cells on Cardiopathy) was a phase 1/2, randomized, double-blind, placebo-controlled clinical trial. The study was conducted at Clínica Santa Maria and Clínica Dávila, Chile. Participants were referred from these private healthcare centers or public hospitals and randomized between December 2012 and June 2014. The experimental design was approved by the ethics committee at both participant health centers and the Chilean Metropolitan Health Service. Before enrollment, all patients agreed to participate and signed an informed consent approved by the institutional review board. This study was registered in Clinicaltrials.gov.

Inclusion criteria were as follows: (1) 18 to 75 years of age, (2) chronic HFrEF with New York Heart Association (NYHA) classification I to III and left ventricular ejection fraction (LVEF) ≤40% at echocardiographic assessment, and (3) all patients had to be under optimal medical management for at least 3 months before randomization, which encompassed class I guideline-recommended therapies (angiotensin-converting enzyme inhibitor or angiotensin receptor blocker, β-blocker, and mineralocorticoid receptor blocker) at maximal tolerable dosages. Ivabradine and sacubtril/valsartan were not included given their recent introduction in our country. Exclusion criteria were as follows: (1) End-stage HFrEF defined as patients with American College of Cardiology Foundation/American Heart Association (ACCF/AHA) stage D (candidates for specialized interventions, including heart transplantation and mechanical assistance) or terminal HF (advanced HF with poor response to all forms of treatment, frequent hospitalizations, and life expectancy <6 months). (2) Recurrent myocardial ischemia defined as any type of acute coronary syndrome 3 months before enrollment. (3) Uncontrolled ventricular tachycardia defined by sustained ventricular tachycardia, including electrical storm and incessant ventricular tachycardia with no response to antiarrhythmic medication. (4) Malignant disease with life expectancy <1 year according to tumor, node, metastasis classification. (5) Manifest ventricular asynchrony defined by intraventricular asynchrony at qualitative echocardiographic assessment (ondulating systolic movement beginning at the interventricular septum and extending to other left ventricular segments, with late activation of left ventricular lateral wall). Patients with left bundle branch block without manifest ventricular asynchrony were allowed to enroll. (6) Hematologic disease: anemia (hemoglobin ≤9.5 g/dL); leukopenia (<4000/μL); thrombocytopenia (<75 000/μL); myeloproliferative disorders, myelodysplastic syndrome, acute or chronic leukemia, and plasma cell dyscrasias (multiple myeloma, amyloidosis). (7) Recent cerebrovascular disease (<3 months). (8) Serum creatinine >2.26 mg/dL (>200 µmol/L). (9) Atrial fibrillation without optimal heart rate control in the last 3 months. Every patient assessed for eligibility was subject to coronary angiography and exercise stress test to guarantee the stability of their coronary disease and rule out signs of ischemia before inclusion into the protocol. Hence, the patients with ischemic cardiomyopathy had predominantly scar.

Eligible patients were enrolled in a 1:1 randomization to intravenous infusion of UC-MSCs or placebo. The randomization list was computer generated by a person unrelated to the study. All patients were assessed at baseline and at the pre-established follow-up points of 3, 6, and 12 months. These evaluations consisted of a clinical assessment for adverse events and NYHA functional classification; Minnesota Living with Heart Failure Questionnaire (MLHFQ) and Kansas City Cardiomyopathy Questionnaire; laboratory testing including complete blood count, liver and renal function tests, brain natriuretic peptide, and high sensitivity C-reactive protein; resting ECG, signal averaged ECG, 24-hour ECG Holter monitoring; transthoracic echocardiography, cardiac magnetic resonance (CMR), and cardiopulmonary exercise test. Technical specifications on quality of life questionnaires, echocardiography, CMR, and cardiopulmonary exercise tests are provided below. Clinical researchers, study nurses, and patients were blinded to treatment allocation.

### Preparation, Characterization, and Infusion of UC-MSC

UC-MSC treatments were processed in an ISO 9001:2015 certified good manufacturing practice type Laboratory (Cells for Cells, Santiago, Chile) under good manufacturing practice conditions according to the Food and Drug Administration Guidance for industry (current good tissue practice) and additional requirements for manufacturers of human cells, tissues, and cellular and tissue-based products. Umbilical cords were obtained from full-term human placentas by caesarean section after informed consent, from healthy donors, and were aseptically stored in sterile PBS supplemented with 100 U/mL penicillin and 100 µg/mL streptomycin (Gibco, Gran Island). Within 3 hours of birth, the umbilical cord was sectioned and washed with PBS and antibiotics. Wharton’s jelly was dissected into small fragments (1–2 mm^2^ pieces), seeded in 100-mm culture plates, and maintained in Minimum Essential Medium Eagle Alpha Modifications high glucose (Gibco, Gran Island) supplemented with 10% heat-inactivated fetal bovine serum (Gibco), 1% penicillin/streptomycin, and 2 mmol/L l-glutamine (Gibco, Gran Island). At 48 hours, nonadherent cells were removed, washed with PBS, and culture medium was replaced with fresh medium every 3 days. When the cell culture reached 70% to 80% confluence, cells were detached by treatment with TrypLE TM Express (Gibco, Gran Island) and reseeded at a density of 2500 cells per cm^2^ into 500 cm^2^ flasks (Nunc, Denmark). At passage 3, UC-MSCs were characterized according to the International Society for Cellular Therapy Guidelines,^15^ harvested, and cryopreserved in Profreeze (Lonza, Walkersville) following the manufacturer’s instruction. In vitro tests (described in the Online Data Supplement) were performed to further characterize the UC-MSCs used in the trial, including cell size and doubling time, senescence markers, cardiomyogenic differentiation potential, paracrine and immunomodulatory activity, and migration capacity of UC-MSCs as compared with BM-MSCs. BM-MSCs were obtained from a 18-year-old healthy male undergoing surgery because of hip trauma, and 2 iliac crest samples that were from a female and a male healthy donor, aged, respectively, 23 and 30 years purchased from Lonza. None had cardiovascular diseases.

According to the amount of cells required in each case, cryopreserved vials were thawed and expanded until passage 5 to 6 using Minimum Essential Medium Eagle Alpha Modifications supplemented with 10% AB plasma. Human leukocyte antigen (HLA) typing for these cells was assessed by polymerase chain reaction for *HLA class I (A, B, C*) and *class II (DP, DQ, DR*). The release criteria for clinical use of UC-MSCs included the absence of macroscopic clumps, contamination by pathogenic microorganisms (bacteria, mycoplasma, syphilis, hepatitis B virus, hepatitis C virus, human immunodeficiency virus, cytomegalovirus, and fungi) or endotoxin (≤0.5 EU/mL), and a viability >80%, with an identity and purity pattern characterized by positivity (≥95%) of CD73, CD90, and CD105 and negative expression (≤2%) of CD45, CD34, CD14, and HLA-DR. A total of 1×10^6^ UC-MSCs/kg of body weight were resuspended in a final volume of 100 mL of AB plasma. The placebo group received 100 mL of autologous plasma. Patients received premedication with intravenous hydrocortisone 100 mg and chlorphenamine 10 mg, complying the local protocol for prevention of allergic and nonhemolytic transfusion reactions. After 30 minutes, they were infused with UC-MSCs or placebo at 2 mL/min via peripheral vein, under noninvasive monitoring of vital signs.

### Study End Points

The primary safety end points encompassed immediate adverse events after intravenous infusion of UC-MSCs or placebo; incidence of overall death, major cardiovascular events (defined by the combined outcome of cardiovascular deaths, hospital admission because of decompensated HF, nonfatal myocardial infarction), and other adverse events, including stroke, sustained ventricular arrhythmias, and incident malignancy. The humoral immune response to infused allogeneic UC-MSCs was tested in a group of 12 patients (7 treated with UC-MSCs, 5 receiving placebo) at days 0, 15, and 90 of infusion using Luminex 200 (Kashi Clinical Laboratories Inc., Portland, OR).

The primary efficacy end point was change in LVEF in echocardiography.^16^ Secondary efficacy end points included changes in left ventricular end-systolic volume (LVESV) and end-diastolic volume (LVEDV) at echocardiography; LVEF, LVESV, and LVEDV in CMR; NYHA functional classification; quality of life questionnaires overall scores; maximum peak oxygen consumption (peak VO_2_) and ventilatory efficiency (VE/VCO_2_ slope) assessed through cardiopulmonary exercise test; brain natriuretic peptide and high sensitivity C-reactive protein.

### Transthoracic Echocardiography

Transthoracic echocardiography was performed by 2 experienced cardiologists, blind from treatment allocation, from both participating centers. Studies were performed in Vivid 7 Dimension Cardiovascular Ultrasound System (General Electric Healthcare). LVEF was measured through modified Simpson biplane method, and LVESV and LVEDV were measured at parasternal long axis in 4 and 2 chambers. Chamber quantifications, diastolic dysfunction, and global longitudinal strain were measured according to recommendations of the American Society of Echocardiography.^17,18^

### Cardiac Magnetic Resonance

CMR studies were performed on a 1.5-Tesla magnetic resonance system using cardiac phased-array SENSE coil with 5 channels (Philips Achieva, The Netherlands). All scans were obtained by a single operator and at a single institution (Clinica Davila, Chile). The imaging protocol included axial, coronal, and sagittal scout images to localize the heart; afterward balanced steady-state free precession (SSFP) cine ECG-gated sequence in 4-chamber, 3-chamber, long axis, and short-axis planes were performed for left ventricular functional assessment. Images were transferred to the workstation (Philips Extended MR Workspace, 2.6.3.5, The Netherlands) for post-processing. Global left ventricular function was quantified by radiologists blinded to treatment allocation, using Segment v1.9 software (Medviso AB, Sweden).^19^ Endocardial and epicardial contours were drawn on short-axis end-diastolic and end-systolic images by radiologists, blinded to treatment allocation and affiliated to an independent institution (Hospital Clinico Universidad de Chile, Chile). Papillary muscles and endocardial trabeculations were included into left ventricular volume. A total of 8 to 12 short-axis segments were needed to encompass the entire left ventricle.

### Cardiopulmonary Exercise Test

Standardized symptom-limited cardiopulmonary test exercise protocols with treadmill or cycle ergometry were performed, based on availability of the technique at each healthcare center of recruitment. Gas exchange measurements analyzed for each breathing cycle were performed using metabolic charts. Exercise capacity variables, including peak VO_2_, VE/VCO_2_, metabolic equivalents (METS), oxygen consumption at anaerobic threshold, peak respiratory exchange ratio, and exercise time, were recorded.

### Quality of Life Questionnaires

Patients answered validated translations of MLHFQ and Kansas City Cardiomyopathy Questionnaire. MLHFQ is a 21-item self-administered questionnaire assessing the patients’ perception of the effects of HF on physical, socioeconomic, and psychological aspects of their life.^20^ Scores range between 0 and 105, and higher scores indicate worst quality of life.^20^ Kansas City Cardiomyopathy Questionnaire is a 23-item self-administered questionnaire addressing specific health domains pertaining to HF: physical limitation, symptoms, quality of life, social limitation, symptom stability, and self-efficacy.^21^ The first 4 domains combine into a clinical summary score. Scores range from 0 to 100, and higher scores point to lower symptom burden and better quality of life.^21^

### Statistical Analysis

Continuous data are expressed as mean±SD and categorical data as absolute number. Categorical data were compared using Pearson χ^2^ test. Continuous data were assessed by Shapiro–Wilk test for normality. Comparison between groups at baseline was assessed through unpaired *t* test or Mann–Whitney *U* test according to normality. Intraindividual comparison of continuous variables at baseline with those at follow-up was performed with paired *t* test or Wilcoxon rank-sum test according to normality. Statistical significance was assumed at a value of *P*<0.05. For comparisons of various post-treatment evaluations versus baseline, Bonferroni α correction was performed, and statistical significance was assumed at a value of *P*<0.0167. CMR studies were additionally analyzed through a mixed effect maximum likelihood regression. In vitro data are expressed as mean±SEM and were compared using 1-way ANOVA followed by Bonferroni correction. A value of *P*<0.05 was considered statistically significant. Analyses were performed with IBM SPSS Statistics 20.0 (IBM Corp) and STATA 12.0 (StataCorp).

## Results

### Characterization of the UC-MSCs

UC-MSCs and BM-MSCs were grown and characterized for surface markers as described above. Their capacity to differentiate to mesodermal lineages was confirmed under specific osteogenic, chondrogenic, and adipogenic differentiation conditions (Online Figure I). Cell size, doubling time, and senescence markers can be seen in the Online Figure II.

### Cardiac Differentiation Potential

**Figure 1.**
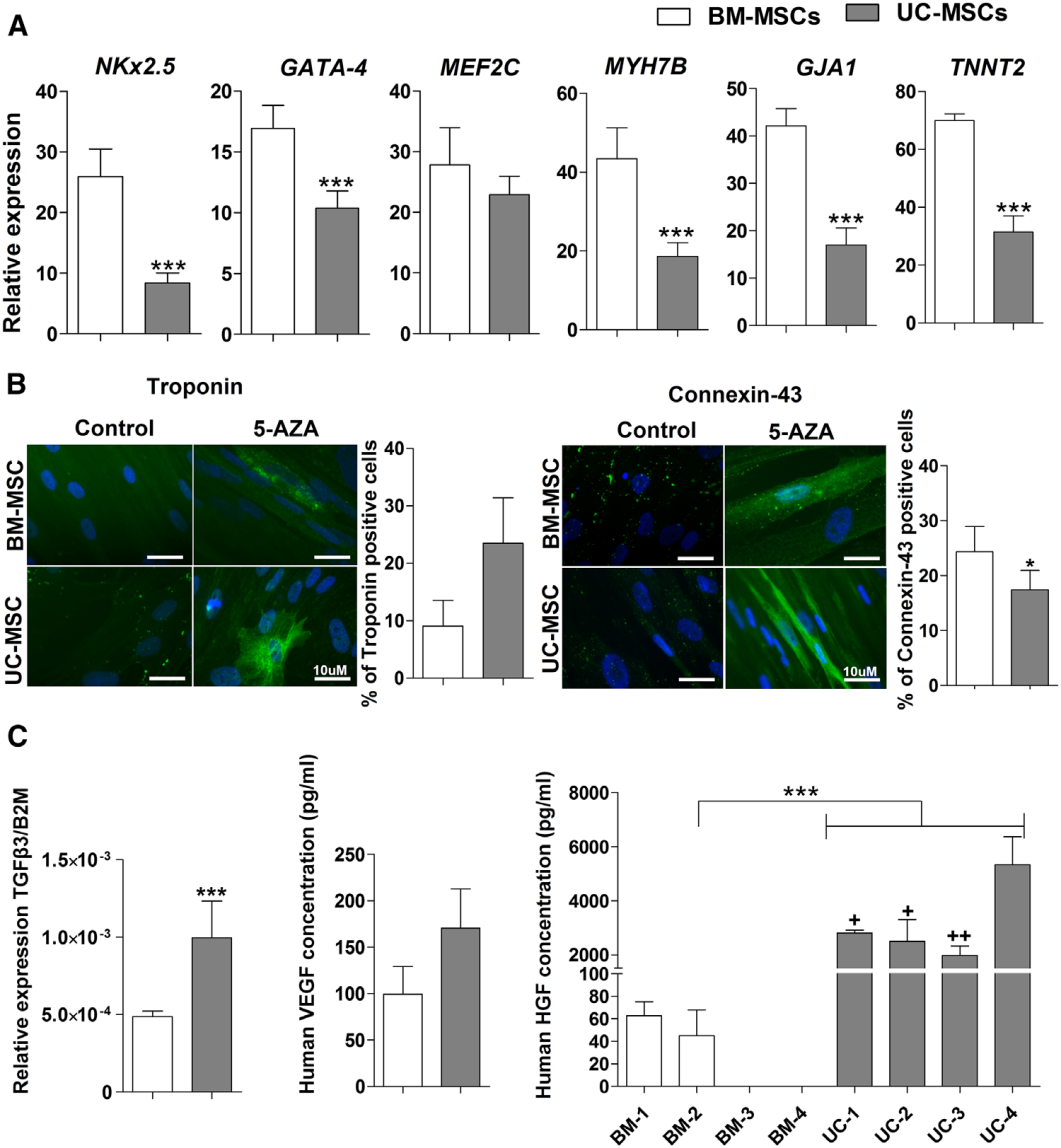
**Umbilical cord–derived mesenchymal stem cells (UC-MSCs) and marrow–derived mesenchymal stem cells (BM-MSCs) displayed different cardiac differentiation potential and paracrine factors profile.** Cardiac differentiation was induced in UC-MSCs and BM-MSCs by cultured with 5-azacytidine (5-AZA) 10 µmol/L during 25 d. Cardiac differentiation potential was evaluated through mRNA relative expression of cardiac gene (*NCx2.5*, *GATA-4*, *MEF2C*, *MYH7B*, *GJA1*, and *TNNT2*) by real time polymerase chain reaction (RT-PCR) with *B2M* as a housekeeping gene (**A**) and by detection of cardiac proteins using indirect immunofluorescence staining troponin and connexin-43 (**B**), the respective graphs show the quantification of positive cells in the each staining. *TGFβ3* expression was quantitated by quantitative RT-PCR (**C**). Vascular endothelial growth factor (VEGF) and hepatocyte growth factor (HGF) levels were evaluated by ELISA assay (**C**). Data shown in the graphs are the mean±SEM of at least 3 individual experiments. *P<0.05, ***P<0.001, UC-MSCs compared with BM-MSCs. +*P*<0.05, ++*P*<0.001 UC-MSC-4 compared with UC-MSCs-1, 2, and 3.

Treatment with 5-azacytidine (5-AZA) for 25 days induced cardiomyogenic differentiation of UC-MSCs, revealed by the expression of specific markers, including transcription factors involved in myogenesis (*NKx2.5*, *GATA-4*, and *MEF2C*) and other genes (*MYH7B*, *GJA1*, and *TNNT2*). The expression of all 6 genes was induced in both cell sources although BM-MSCs exhibited higher mRNA levels as shown by reverse transcription polymerase chain reaction (*P*<0.001; Figure 1A). Conexin-43 staining also had greater expression in BM-MSCs than in UC-MSCs (24.33±1.84% versus 17.42±1.43%; *P*=0.018). In contrast, troponin expression seemed increased in UC-MSCs (23.47±7.94% versus 9.06±2.61% for BM-MSCs) but did not reach significance (*P*=0.166; Figure 1B). Beating was not observed in MSCs after induction with 5-AZA.

### Paracrine Profile

UC-MSCs showed a higher transforming growth factor beta 3 (TGF-β3) gene expression in comparison with BM-MSCs (*P*<0.001), but vascular endothelial growth factor expression levels in comparison with BM-MSCs was not significantly different (Figure 1C). Of note, UC-MSCs showed a 55-fold higher expression of hepatocyte growth factor in comparison with BM-MSCs (*P*>0.0001; Figure 1C), that in some cases showed undetected levels of hepatocyte growth factor. Comparative quantification of indoleamine 2 3-dioxygenase (IDO) activity, interleukin 6 (IL6), TGF-β1, prostaglandin E2 (PGE2), HLA-G, and programmed death-ligand 1 (PD-L1) at basal and stimulated condition of UC-MSCs and BM-MSCs can be seen at Online Figure III.

### Immunomodulatory Effects

**Figure 2.**
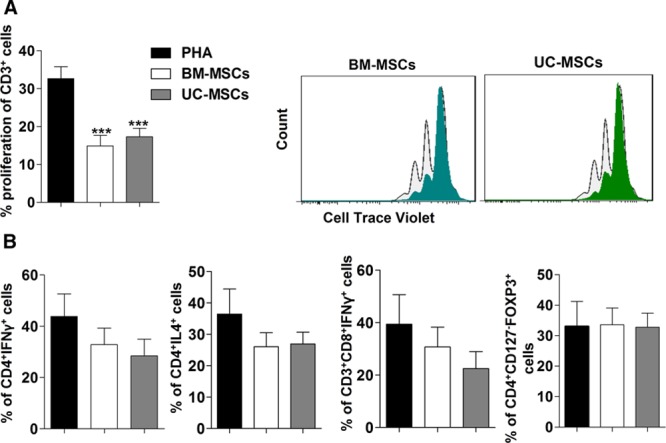
**Umbilical cord–derived mesenchymal stem cells (UC-MSCs) and marrow–derived mesenchymal stem cells (BM-MSCs) display the same suppressive capacities to inhibit proinflammatory T-cells.** PHA-activated peripheral blood mononuclear cells (PBMC) obtained from dilated cardiomyopathy patients with heart failure and reduced ejection fraction (HFrEF) labeled with 5(6)-carboxyfluorescein diacetate N-succinimidyl (CFSE) were coculture with or without mesenchymal stem cells (MSCs) at a 1:10 ratio (MSCs:PBMC). **A**, T-cell proliferation was evaluated by the reduction in CFSE intensity at 72 h after culture, the graphs in the left is a representative CFSE proliferation panel (light color histogram represents activated PBMCs and dark color histogram to activated PBMC cocultured with MSCs). **B**, Th1, Th2, CD8, and regulatory T cells subsets analysis from coculture of PBMC and MSCs. Results are represented as mean±SEM of at least 3 independent experiments using at least 3 different donors for PBMC (healthy donor and HF patient), UC-MSCs, and BM-MSCs. ***P<0.001 UC-MSCs or BM-MSCs with respect to PHA.

The immunosuppressive properties of UC-MSCs were assessed by evaluating their effect on the proliferative response of peripheral blood mononuclear cells (PBMC) after phytohemagglutinin (PHA) stimulation in vitro. UC-MSCs exhibited a similar inhibitory effect on T-cell proliferation compared with BM-MSCs at the 1:10 ratio, and inhibition percentages were 21.53±3.85% and 23.96±4.50% for UC-MSCs and BM-MSCs, with respect to PHA-induced proliferation in the absence of MSCs (*P*<0.005 versus control; Figure 2A). T helper 1, T helper 2, and cytotoxic T cells exhibited a tendency to decrease their proliferation after cocultured with UC-MSCs or BM-MSCs (*P*>0.05). No effect of the MSC cocultures was observed on regulatory T-cell proliferation (Figure 2B).

### Migration Profile in Response to HFrEF Patient’s Serum

**Figure 3.**
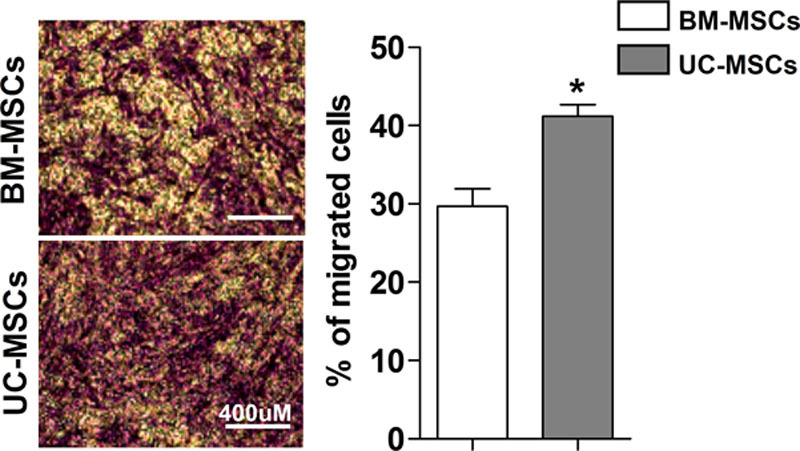
**Umbilical cord–derived mesenchymal stem cells (UC-MSCs) possess a superior migration capacity compared with marrow–derived mesenchymal stem cells (BM-MSCs).** Migration capacity of MSCs was evaluated by transwell assay in response to serum from patients with heart failure and reduced ejection fraction after 16 h. The pictures show the representative staining with violet crystal and the left graph the quantification of % of migrated cells under the different conditions. Data shown in the graphs are the mean±SEM of at least 3 serum donors, UC-MSCs, and BM-MSCs. *P<0.05 UC-MSCs vs BM-MSCs.

The percentage of migrating cells was significantly higher in UC-MSCs compared with BM-MSCs in response to HFrEF patient’s serum (41.18±6.53% versus 29.67±8.35%; *P*<0.01; Figure 3).

### Patient Population

**Figure 4.**
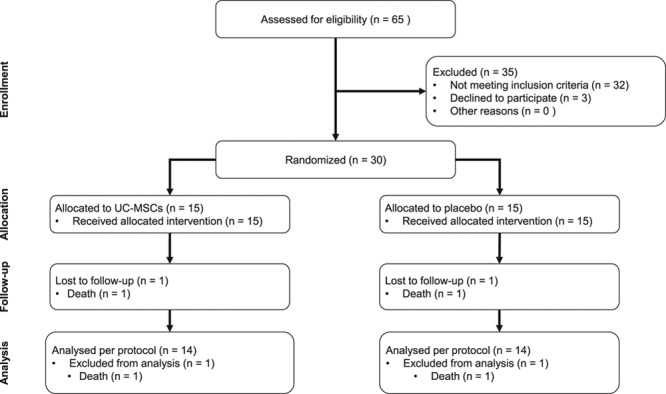
**Study flow chart**. UC-MSC indicates umbilical cord–derived mesenchymal stem cell.

From December 2012 to June 2014, 65 patients were assessed for eligibility, 30 patients underwent randomization (n=15 per group; Figure 4). Baseline characteristics of the UC-MSC and placebo groups did not differ in terms of demographic variables, cardiovascular risk factors, NYHA class, and electrocardiography (Table 1). Ischemic cardiomyopathy was the predominant pathogenesis of HFrEF (21 patients, 70%). There were no differences between groups concerning therapeutic agents that modify cardiac remodeling. No patient had cardiac implantable electronic devices. One patient from each group had left bundle branch block although none presented manifest ventricular asynchrony at baseline. Patients treated with placebo presented higher brain natriuretic peptide levels and 25% greater LVEDV at baseline (*P*<0.05).

**Table 1.**
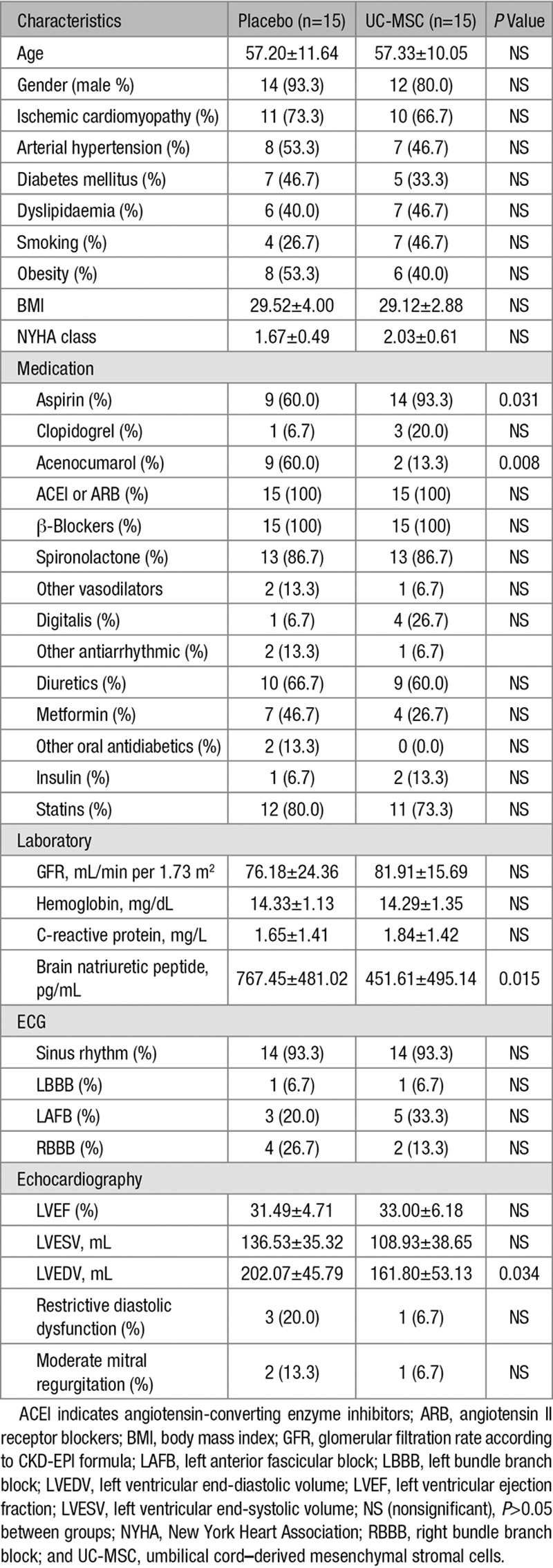
Baseline Characteristics

### Safety

There were no acute adverse events associated with the infusion of allogenic UC-MSCs or placebo. None of the tested individuals (7 treated with UC-MSC and 5 receiving placebo) developed alloantigen-directed antibodies post-infusion. Of note, 1 female patient with baseline reactivity to 52 different HLA specificities before UC-MSC treatment lost reactivity to 16 of these specificities at day 90. Furthermore, because we typed the infused cells, we could detect that only 21% of specificities not expressed on the infused MSCs disappeared, as opposed to 100% of those present on the infused MSCs (*P*=0.004). Our data not only confirm the absence of humoral immune reaction to UC-MSCs but also suggest that MSCs preferentially suppress reactivity to their own HLA molecules.

Clinically relevant events throughout the 12 months of follow-up are shown in Table 2. The deceased patient from the placebo group had an acute myocardial infarction at 5 months of follow-up. The patient from the UC-MSC group presented an acute lymphocytic leukemia at 5 months from intravenous infusion of UC-MSC, lacking clinical and laboratory elements suggestive of leukemia at baseline and at 3 months of follow-up. One patient from the placebo group developed a malignant melanoma. Concerning major cardiovascular events, 3 patients from the placebo group and 1 from the UC-MSC group had hospitalizations because of decompensated HF, only 1 patient experienced an acute coronary syndrome in the placebo group. None of the patients had an acute ischemic stroke. No new-onset supraventricular arrhythmias, sustained ventricular arrhythmias, atrioventricular blocks, or bundle branch blocks were diagnosed during follow-up, and none were observed at ECG Holter monitoring. There was an increase in the amount of premature ventricular complexes at 24-hour ECG monitoring in the placebo group at follow-up albeit without changes in mean Lown classification (Online Table I). No noteworthy variations were observed in time or frequency domains at follow-up. No thoracic ectopic tissue formation was observed in CMR at completion of this study. No significant abnormalities were seen in complete blood counts, renal and liver function during monitoring points.

**Table 2.**
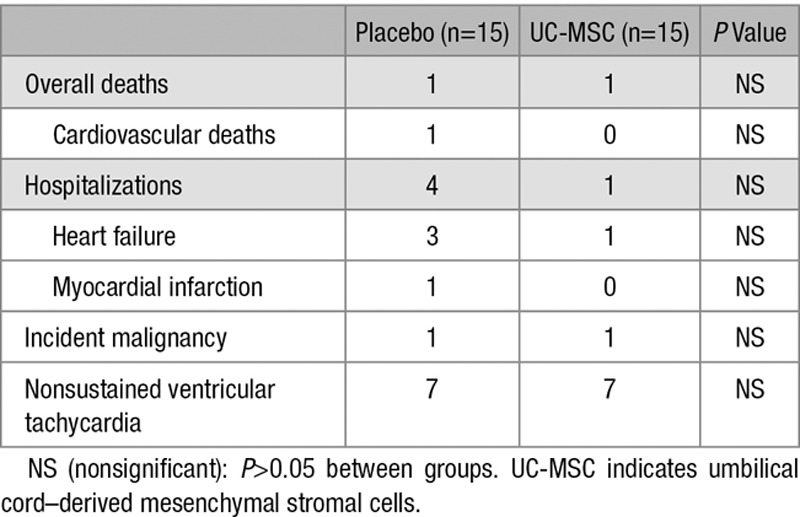
Incidence of Clinically Relevant Events at 12-Month Follow-Up

### Cardiac Imaging

Echocardiographic parameters evaluated at baseline and follow-up are depicted in Table 3. Compared with baseline, there were improvements in LVEF in the UC-MSC–treated group that began at 3 months of follow-up (+3.71±5.01%; *P*=0.010) and continued at 6 months (+5.43±4.99%; *P*=0.001) and 12 months (+7.07±6.22%; *P*=0.001). There were no changes in left ventricular volumes. The placebo group showed no major differences in these variables. The change of LVEF from baseline to month 12 differed significantly for both groups (+7.07±6.22% versus +1.85±5.60%; *P*=0.028).

**Table 3.**
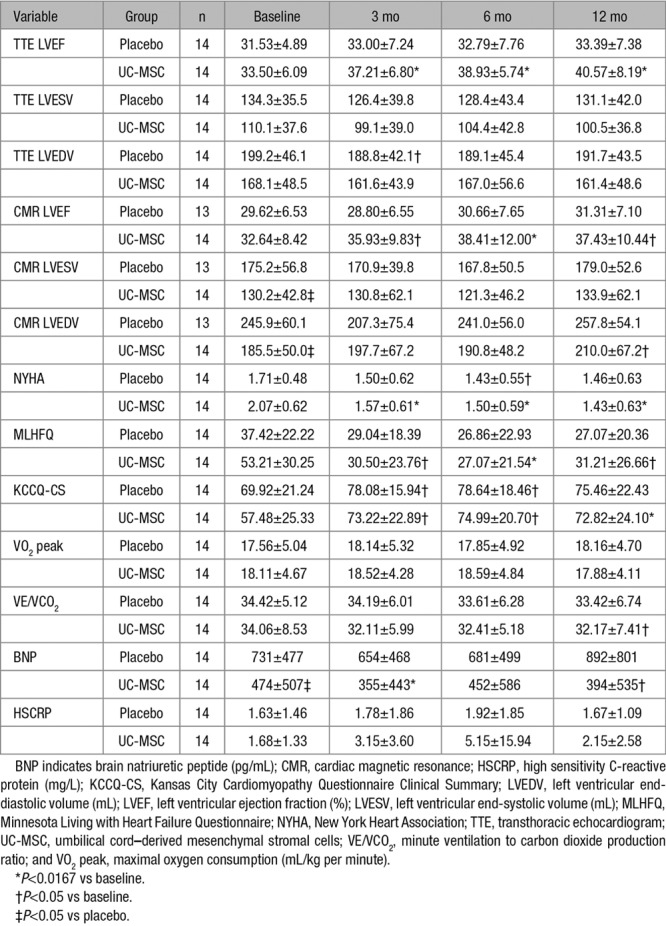
Primary and Secondary Efficacy Outcomes at Baseline and Follow-Up Points

**Figure 5.**
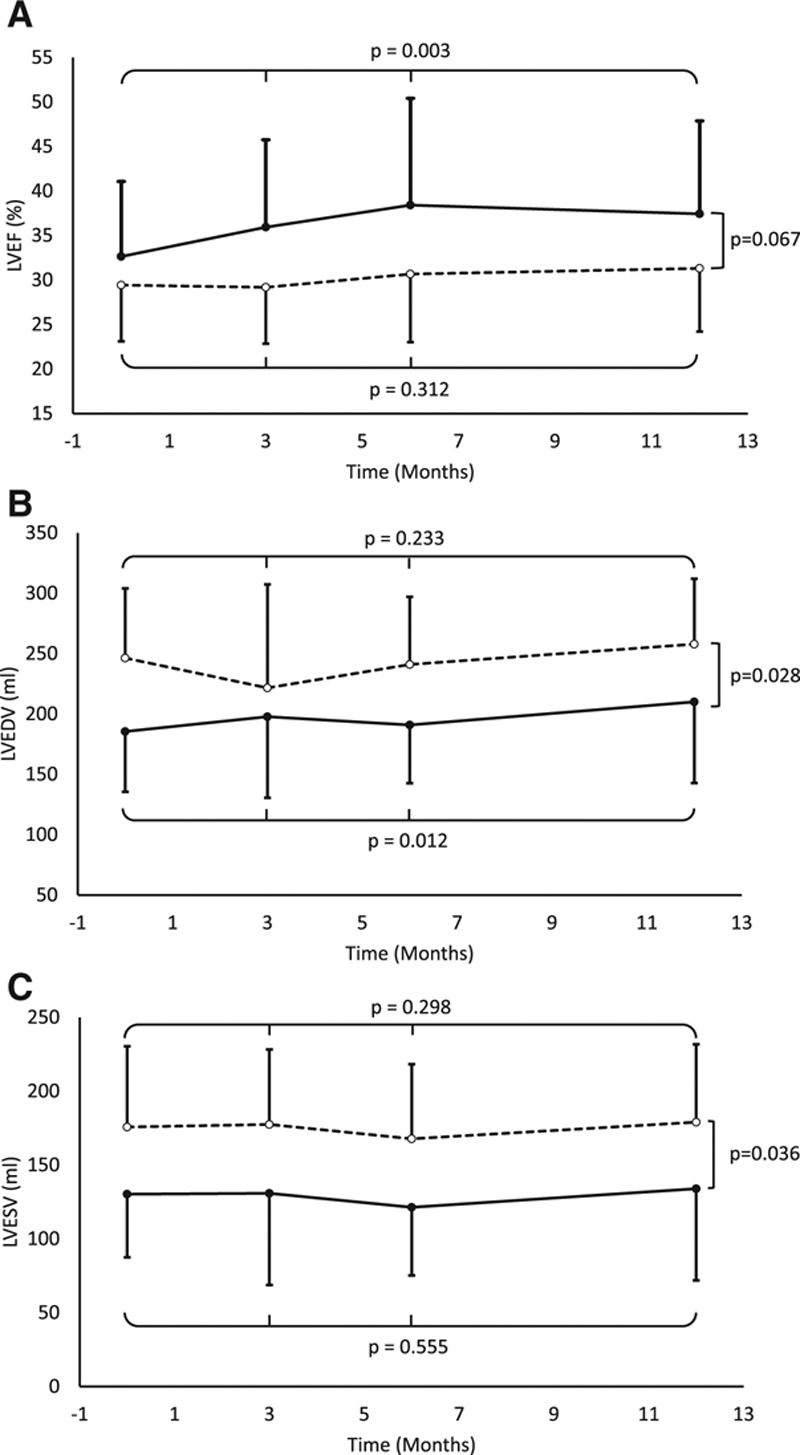
**Changes in CMR** **measurements from baseline to 12**-**mo** **post**-**treatment in studied groups.** **A**, Left ventricular ejection fraction (LVEF). **B**, Left ventricular end-diastolic volume (LVEDV). **C**, Left ventricular end-systolic volume (LVESV). Continuous line represents umbilical cord–derived mesenchymal stem cell group (n=14 per protocol). Dashed line represents placebo group (n=13 per protocol; withdrawal of consent from 1 patient). Statistical analysis is based on mixed effect maximum likelihood regression between baseline and follow-up measures for each group and variability between groups.

CMR measurements are shown in Table 3. Patients treated with intravenous infusion of UC-MSCs presented an increase of LVEF (*P*=0.0003) and LVEDV (*P*=0.012; Figure 5). The most significant improvements of LVEF was at 6 months of follow-up (+4.67±4.51; *P*=0.005). There was an increase in LVEDV in the UC-MSC group at 12 months (*P*=0.033). We observed no changes in LVEF or left ventricular volumes in the placebo group (n=13). One patient from the placebo group withdrew consent for CMR.

### Functional Status, Quality of Life, and Clinical Biomarkers

Results are summarized in Table 3. There were substantial improvements in NYHA class in patients treated with UC-MSCs, starting at 3 months (−0.54±0.56; *P*=0.011), which remained at 12 months follow-up (−0.62±0.46; *P*=0.003). Only the UC-MSC group experienced improvements in MLHFQ from baseline to all follow-up points (*P*<0.05). Both groups experienced an initial improvement of Kansas City Cardiomyopathy Questionnaire clinical summary at 3 and 6 months of follow-up, with persistence of improvement at trial completion only in the UC-MSC–treated group (*P*=0.014). Patients treated with UC-MSCs exhibited an improvement in VE/VCO_2_ at 12 months (−1.89±3.19; *P*=0.023 versus baseline) while no differences were observed in peak VO_2_. We found no differences in other exercise capacity variables, including METS, oxygen consumption at anaerobic threshold, peak respiratory exchange ratio, and exercise time after cell therapy (Online Table II). We observed a slight decrease in brain natriuretic peptide levels in the group treated with UC-MSCs at 3 and 12 months of follow-up.

## Discussion

RIMECARD is the first randomized, double-blind, placebo controlled clinical trial with intravenous infusion of allogenic UC-MSCs in patients with chronic HFrEF. Intravenous infusions of UC-MSCs are safe in this population and suggest benefits in surrogate clinical end points, including LVEF, functional status, and quality of life, in patients with HFrEF receiving this form of systemic stem cell therapy.

MSC-based therapies have been considered overall safe procedures. A recent systematic review of 36 prospective clinical trials for several clinical conditions, including myocardial infarction and chronic cardiomyopathy, did not detect an association between intravascular infusions of MSCs and the risk of acute infusion toxicity, organ system complications, infection, death, or malignancy in treated patients.^22^ Systematic reviews in HF population actually describe an association between stem cell therapy and a reduction of mortality and major cardiovascular events albeit most of the analyzed studies used intramyocardial injection or percutaneous intracoronary infusion of bone marrow mononuclear cells.^4,5^ There is limited experience on intravenous administration of MSCs in patients with cardiovascular diseases, mainly because of safety concerns on the entrapment of donor cells in pulmonary circulation and apprehensions on their therapeutic efficacy in a context of low cardiac engraftment. A phase 2 study by Hare et al^23^ supports the safety of intravenous administration of allogenic BM-MSCs (up to 5×10^6^ cells/kg) in acute myocardial infarction. At 6 months of follow-up, MSC-treated patients had similar adverse event rates, a trend toward decreased in hospitalization rate and a decrease in arrhythmic events versus placebo.^23^ In addition, there were benefits in pulmonary function at 6 months and lack of evidence of pulmonary ectopic formations in CMR studies performed at 12 months.^23^ A recent crossover phase 2 clinical trial by Butler et al^24^ assessed the safety of the intravenous administration of ischemia-tolerant allogenic BM-MSCs versus placebo in patients with nonischemic cardiomyopathy. At 90 days of follow-up, this trial reported no differences in death, hospitalizations, and serious adverse events between groups.^24^ Considering both studies and our results, the intravenous delivery of UC-MSCs seems safe in HFrEF population. Intravenous infusion of UC-MSCs was not associated with a decrease in the incidence of ventricular arrhythmias, unlike the study by Hare et al^23^; a difference that could be because of several reasons including different patient populations, MSC dosages, and monitoring time points.

Our trial displayed improvements in LVEF in patients receiving intravenous UC-MSC treatment albeit no noteworthy reductions in LVESV or LVEDV were observed. Randomized clinical trials with autologous and allogenic MSCs have reported differing results on evolution of left ventricular systolic function and volumes.^24–32^ In the dose-escalation POSEIDON trial (Percutaneous Stem Cell Injection Delivery Effects on Neomyogenesis Pilot Study), patients with ischemic HF who received transendocardial injections of autologous and allogenic BM-MSCs showed nonclinically relevant improvement on LVEF within 13 months (mean increase +1.96%; *P*=0.11; n=27).^25^ In the later POSEIDON-DCM trial (Percutaneous Stem Cell Injection Delivery Effects On Neomyogenesis in Dilated Cardiomyopathy), a phase I/II randomized clinical trial in patients with nonischemic dilated cardiomyopathy comparing transendocardial injections of allogenic versus autologous BM-MSCs (100×10^6^ cells), an increase in LVEF was described for patients receiving allogenic BM-MSCs at 12 months (+8.0%; *P*=0.004; n=18) while patients with autologous BM-MSCs exhibited nonsignificant changes (+5.4%; *P*=0.116; n=16); there were no changes in ventricular volumes for both groups.^30^ In the C-CURE trial (Cardiopoietic Stem Cell Therapy in Heart Failure), the group of patients with ischemic HFrEF treated with a combination of autologous BM-MSCs exposed to a cytokine cocktail for cardiogenic differentiation (mean dose, 733×10^6^ cells; n=32), presented noteworthy improvements in LVEF (+6.8%; *P*<0.0001) and LVESV (−16 mL; *P*<0.0001) at 6-month follow-up.^26^ In the TAC-HFT (Transendocardial Autologous Cells in Ischemic Heart Failure Trial), patients with ischemic HF receiving intramyocardial injections of autologous BM-MSCs (100–200×10^6^ cells; n=19) showed nonsignificant trends toward improvement in LVEF, LVESV, and LVEDV at 12 months.^27^ In the MSC-HF trial (Bone Marrow-Derived Mesenchymal Stromal Cell Treatment in Patients With Severe HF), patients with ischemic HF receiving intramyocardial injections of autologous BM-MSCs (mean dose, 77.5×10^6^ cells; n=40) exhibited an increase in LVEF (+5.0%; *P*<0.0001) and a decrease in LVESV (−7.6 mL; *P*=0.001) while no changes in LVEDV were observed at 6-month follow-up^28^. A phase 2 dose-escalation study in patients with HFrEF performed by Perin et al,^32^ assessing transendocardial injections of immunoselected allogenic BM-MSCs (25, 75, and 150×10^6^ cells; n=15 per group), revealed no differences in LVEF at 12 months of follow-up although the 150×10^6^ MSC group had a significant reduction in LVESV and LVEDV at 6 months and a nonsignificant decrease of both ventricular volumes at 12 months. In a randomized trial by Zhao et al^29^ in patients with decompensated HFrEF, individuals receiving intracoronary injections of allogenic UC-MSCs (n=30) presented improvements in LVEF (+19.0±6.8%; *P*<0.01) and LVESV (−13.14±10.62 mL; *P*<0.05) at 6 months. In the recent trial by Butler et al,^24^ patients with HFrEF receiving ischemia-tolerant allogenic BM-MSCs (1.5×10^6^ cells/kg, n=10) experienced a significant increase in LVEF (+2.31%; *P*=0.02) and reductions in LVEDV (−17.86 mL; *P*=0.04) and LVESV (−16.60 mL; *P*=0.02) at 3 months. Remarkably, the ixCELL-DCM trial (Transendocardial Injection of Ixmyelocel-T in Patients With Ischemic Dilated Cardiomyopathy) reported a reduction in the combined outcome of all-cause mortality and cardiovascular admissions (relative risk [RR], 0.63; 95% confidence interval, 0.42–0.97; *P*=0.0344) in patients with symptomatic HFrEF receiving transendocardial injections of ixmyelocel-T (n=58), a multicellular therapy produced from autologous bone marrow mononuclear cells—with selective expansion of MSCs and macrophages—versus placebo (n=51).^31^ These patients receiving ixmyelocel-T experienced no change in LVEF or ventricular volumes.^31^ The CHART-1 trial (Congestive Heart Failure Cardiopoietic Regenerative Therapy) showed neutral results on composite and individual outcomes, including all-cause mortality, worsening HF events, and surrogate end points (LVEF, LVESV, LVEDV, and MLHFQ), in HFrEF patients with ischemic cardiomyopathy receiving intramyocardial injections of cardiopoietic cells (MSCs; n=120) versus sham procedures (n=151).^33^ Exploratory analysis from CHART-1 suggests a benefit in treated individuals with baseline LVEDV >200 mL; unlike our trial, in which most treated patients had lower baseline LVEDV. A recent retrospective cohort of 2166 outpatients with HF by Kalogeropoulos et al concluded that patients who experienced recovery of LVEF (defined as current LVEF >40% but any previously documented LVEF ≤40% by transthoracic echocardiography) had fewer all-cause mortality (RR, 0.71; 95% confidence interval, 0.55–0.91), cardiovascular hospitalizations (RR, 0.50; 95% confidence interval, 0.35–0.71), and HF-related hospitalization (RR, 0.48; 95% confidence interval, 0.30–0.76) compared with patients with HFrEF or HF with preserved LVEF.^34^ In the POSEIDON-DCM trial, such recovery of LVEF was achieved by 46.7% of patients receiving allogenic BM-MSCs and 22.2% of patients treated with autologous BM-MSCs,^30^ whereas in our study, this occurred in 50% (7 of 14) of UC-MSC–treated individuals versus 7.1% (1 of 14) of the placebo group at month 12 (*P*=0.0365). Albeit ours is a small series, only the UC-MSC–treated group exhibited significant improvements in LVEF at 3, 6, and 12 months of follow-up, both by transthoracic echocardiography (*P*=0.0167 versus baseline) and CMR (*P*=0.025 versus baseline). This suggests that our patients might experience benefits on major clinical outcomes although this observation requires verification in a larger phase 3 clinical trial.

Improvements in NYHA and quality of life questionnaires were observed in the UC-MSC group also, in agreement with results from other MSC-based therapy clinical trials in HF.^24–28,30^ Interestingly, at 12 months of the POSEIDON-DCM trial, the groups receiving allogenic BM-MSCs had 66.7% of patients with improved NYHA functional class and a substantial decrease in mean MLHFQ scores, while patients receiving autologous BM-MSCs exposed only a trend toward improvement.^30^ We appreciated a low concordance between improvement on NYHA classification and performance at cardiopulmonary exercise test, a phenomena previously described.^35^ Cardiopulmonary exercise tests have been seldom performed in cell therapy trials and with wide-ranging results. Regarding MSCs therapies, to our knowledge, only the POSEIDON and TAC-HFT assessed peak VO_2_, describing no changes for this outcome in patients treated with autologous BM-MSCs.^25,27^ We did not observe changes in this variable although we identified a modest improvement in ventilatory efficiency in patients treated with UC-MSCs at 12 months. Recent evidence suggests that VE/VCO_2_ is an excellent marker of severity and prognosis of HF, better than peak VO_2_ at reflecting the complex interplay of pulmonary, cardiac, and peripheral manifestations in HF population.^36,37^ The lack of major benefits in cardiopulmonary performance can be attributed to several factors. Honold et al^38^ in a subanalysis of patients with poor, moderate, and conserved cardiopulmonary test results before cell therapy documented that patients with lowest initial exercise capacity showed largest improvements in peak VO_2_ and VE/VCO_2_ after intracoronary stem cell infusion. Our patients had slight alterations at baseline, therefore limited benefits could be anticipated.

A range of mechanisms have been proposed to explain the clinical benefit observed in patients with HF treated with MSCs, including reductions in myocardial cell apoptosis, modulation of inflammation, myocardial fibrosis, neovascularization, and increased cell differentiation.^13^ Incorporation of MSCs into tissues is regulated by multiple processes, including cell recruitment, migration, and adhesion.^39^ The higher migration of UC-MSCs in response to HFrEF patient serum, herein described, is compatible with the notion that this cell type might sense biological cues that are contributory to their therapeutic effect by systemic delivery.

In our study, UC-MSCs and BM-MSCs expressed cardiomyogenic differentiation potential although BM-MSCs presented a more favorable profile of transcription factors related to cardiac differentiation. Despite early reports describing cell engraftment and differentiation in animal models of HF, later studies evidence retention rates <0.5% after 4 days of intramyocardial injections of BM-MSCs,^40^ which seem insufficient to account for the magnitude of clinical benefit. Mounting evidence rather suggests the reparative actions of MSCs rely on paracrine modulation.^1,2^ The comparative results of the paracrine factors assessed in this work point to a significant advantage of UC-MSCs over BM-MSCs. The most striking difference was the prominent expression of hepatocyte growth factor in UC-MSCs from all tested donors while BM-MSCs showed low to undetectable levels. Remarkably, several studies in chronic ischemic or nonischemic HF animal models have reported that gene transfection of hepatocyte growth factor promotes angiogenesis and decreases fibrosis and apoptosis, attenuating cardiac remodeling and improving myocardial remodeling, perfusion, and contractile function.^41–45^ Furthermore, MSCs share several biological properties with endothelial cells, enabling them to contribute to angiogenesis. Preclinical data from several groups including ours suggest that UC-MSCs can enhance angiogenesis by promoting the formation of capillary-like structures in vitro or increasing capillary density in vivo through upregulation of various proangiogenic factors and chemokines, including vascular endothelial growth factor, angiopoietin, and monocyte chemoattractant protein-1 among others.^12,13,46,47^ Liu et al^14^ have described that intracoronary and intravenous infusion of UC-MSCs was associated with a promotion of angiogenesis through paracrine modulation and perhaps endothelial cell differentiation, an augmented myocardial perfusion and enhancement of collateral vessel development in a porcine model of a chronic myocardial ischemia. In the same study, animals treated with UC-MSCs had improved LVEF and a reduction of myocardial fibrosis and apoptosis.^14^ Moreover, allogenic MSCs can improve endothelial function and vascular reactivity through stimulation of endothelial progenitor cell mobilization in patients with HF.^30,48^ Interestingly, the PROMETHEUS trial (Prospective Randomized Study of Mesenchymal Stem Cell Therapy in Patients Undergoing Cardiac Surgery) assessed the impact of intramyocardial injections of autologous BM-MSCs into the akinetic nonrevascularized myocardial segments of patients with chronic ischemic cardiomyopathy, reporting an improvement in myocardial perfusion and functional recovery, and subsequently an improvement in global left ventricular function.^49^

### Limitations

The assessment of differences in major cardiovascular outcomes and surrogate efficacy outcomes was underpowered because of the small number of participants from each patient group. Post hoc analysis considering echocardiographic assessment of LVEF at 12 months revealed an estimated power of 71%. This discouraged further analysis to discriminate responders from nonresponders to therapy or differences on cardiomyopathy substrate. Differences in left ventricular volumes at baseline, in spite of randomization, could bias efficacy results in favor of UC-MSC. However, the subanalysis of CHART-1 is reassuring in that most benefit in response to treatment occurred precisely in patients with higher baseline LVEDV, suggesting that such bias might not be in favor of our UC-MSC group. We could not perform myocardial perfusion and fibrosis measurements because of noncontrast CMR imaging and software restraints, nonetheless these had not been considered as secondary end points of the study.

### Conclusions

Intravenous infusion of UC-MSCs was feasible and safe in this group of patients with HFrEF under otherwise optimal medical therapy. Allogenic UC-MSC treatment induced no humoral immune response in tested individuals. The intervention resulted in a significant improvement in left ventricular function, functional status, and quality of life. These findings suggest that UC-MSCs could have an impact on clinical outcomes, supporting further testing through large clinical trials.

## Acknowledgments

We thank all participants of this trial and cardiologists who referred patients for the study and technicians from the Laboratory of Nano-Regenerative Medicine and the radiology departments from Clínica Dávila and Universidad de Chile for their technical support. Special thanks to Gabriel Cavada, PhD, for his statistical advice; Flavio Carrión, PhD, for his contribution during the setting up of our GMP Lab; Dr Jesús Herreros, MD, for his initial support for our Program of Translational Research in Cell Therapy; and Paola Fuentes for her secretarial assistance.

## Sources of Funding

This study was supported by a grant from the Chilean Economic Development Agency (CORFO 11IEI-9766).

## Disclosures

P.L. González and F. Alcayaga-Miranda received stipends from Cells for Cells. M. Khoury is CSO of Cells for Cells and Consorcio Regenero. The other authors report no conflicts.

## Supplementary Material

## Novelty and Significance

### What Is Known?

- Intracoronary and intramyocardial cell therapy, mainly with allogenic bone marrow-derived mesenchymal stromal cells (BM-MSC), has shown to be safe and potentially effective in patients with heart failure, even if low levels of cell engraftment are expected, suggesting a paracrine mechanism of action.
- Umbilical cord–derived mesenchymal stromal cells (UC-MSC) are of easier access and in vitro expansion and exhibit superior angiogenic and paracrine effects compared with BM-MSC, but their systemic administration in human heart failure patients has not been tested.
