# Supplementary material for: Safety and Efficacy of the Intravenous Infusion of Umbilical Cord Mesenchymal Stem Cells in Patients With Heart Failure: A Phase 1/2 Randomized Controlled Trial (RIMECARD Trial [Randomized Clinical Trial of Intravenous Infusion Umbilical Cord Mesenchymal Stem Cells on Cardiopathy])
Source: Circ Res. 2017 Oct 26;121(10):1192–204. doi: 10.1161/CIRCRESAHA.117.310712 (PMC6372053; doi:10.1161/CIRCRESAHA.117.310712)
Supplement: Supplementary file 1 [file res-121-1192-s001.pdf]

ONLINE SUPPLEMENT

EXPANDED METHODS

Automated cell counting, size and characterization:

Prior to the release of each batch of cell-therapy, preparations were assessed using a Countess™ (Invitrogen, USA) automated cell counter for viability, cell count and size measurements using trypan blue staining for viability combined with advanced image analysis. After setting the cell mode parameters for MSC cell type (circularity, maximum and minimum cell size), UC-MSC cell samples were mixed with trypan blue (1:1) and loaded onto the cell counting chamber slide. Each sample data was stored and then analyzed.

Doubling time

Data were collected from each cell culture process performed under GMP conditions. Doubling time was calculated based on initial cell number, culture time (hours) and final number of cells harvested according to the described formula (Roth V. 2006 Doubling Time Computing, available from: http://www.doubling-time.com/compute.php): Doubling Time = duration x log (2) / [log (final concentration) −log (initial concentration)]. The range of analyzed data was the following:

Cell seeding density: 1400 – 4900 cells/cm2
Initial cell number seeded: 1.4 – 62 million
Culture days: 3-8 days
Harvested cell number: 8.5 – 336 millions

Senescence-associated beta-galactosidase assay

UC-MSC and BM-MSC were cultured and harvested under standard conditions. To perform the assay using Senescent cells histochemical staining kit (sigma #CS0030), 10.000 cells per well were seeded in 24-well plates. After 5hrs, the staining mixture was added for detection of SA-β-galactosidase and incubated overnight at 37°C following the manufacturer's instructions. Positive stained cells were counted and the percentage of cells expressing SA-β-galactosidase (senescent cells) calculated over the total cell number of each sample.

Cardiomyogenic differentiation

For cardiomyogenic differentiation, cells in passage 3 were seeded at 60% of confluence and cultured in Dulbecco's modified Eagle's medium (DMEM) high glucose (Gibco, USA) supplemented with 5% heat-inactivated FBS (Gibco, USA) with 1% penicillin/streptomycin and 2 mM L-G and 10 uM 5-azacytidine (Sigma-Aldrich, USA). The media was removed and replaced for fresh media every 3 days. At day 20 relative expression of NKX2-5, GATA-4, MEF2C, MYH7b, GJA1 and TNNT2 were measured. RNA extraction was performed using the RNeasy mini kit (Qiagen, USA) and complementary DNA was synthesized in a 20 µl reaction mixture using SuperScript III First-Strand Synthesis for RT-PCR (Invitrogen, USA). RT-qPCR was performed using SYBR Green Reagents (QPCR Master Mix, Agilent Technologies). All primer sets were previously screened for efficiency and their sequences were: B2M (F:5´ TCAGGTTTACTCACGTCATCC 3´, R:5' ACACGGCAGGCATACTCATC 3´), GATA-4 (F: 5´AAACGGAAGCCCAAGAACCT 3´, R: 5´ ACTGAGAACGTCTGGGACAC 3´), NKX2-5 (F: 5´ TGTCCACGCTGCATGGTATC 3´, R:5´GATCACTCATTGCACGCTGC3´), MEF2C (F: 5´ CCAACTTCGAGATGCCAGTCT 3´, R:5´ GTCGATGTGTTACACCAGGAG 3´), MYH7B (F:5'

GCAATAAAAGGGGTAGCAGAGC 3', R:5'GACTCCCCAAGTTCACTCACAT3'), TNNT2 (F:5' CTGGCCATTGACCACCTGAA 3', R:5'GCTGCTTGAACTTCTCCTGC3'), GJA1 (F:5' TCTCTCATGTGCGCTTCTGG 3', R:5' TGACACCATCAGTTTGGGCA 3'). Data were expressed as relative mRNA level of specific gene using the 2-ΔΔCT method and normalized with the Beta-2 microglobulin (B2M) housekeeping gene. Additionally, differentiation was confirmed by indirect immunofluorescence using Anti-Cardiac Troponin I antibody (ab47003, Abcam), Anti-Connexin 43/GJA1 (ab47368, Abcam) and Goat Anti-Rabbit IgG FITC (ab6717, Abcam). Cells were both fixed with 4% paraformaldehyde (PFA) for 10 minutes at room temperature and permeabilized with 0.3% Triton X-100 for 10 minutes. Nonspecific binding was blocked by PBS plus 5% serum albumin bovine serum (BSA) (Sigma) for 60 minutes. Primary antibodies were incubated overnight at 4°C and fluorescein isothiocyanate (FITC)-conjugated secondary antibodies for 2 hours at room temperature. Nuclei were counterstained with 4´, 6´diamino-2-phenylindole (Sigma) for 1 minute. Images were obtained with a NIKON ECLIPSE TE2000-fluorescence microscopy and Nikon Sight DSU2 camera.

Measurements of Paracrine Factors

To compare the secretion levels of growth factors between BM–MSCs and UC–MSCs, $3X10^4$ cells were plated in serum-free medium in 6-well plates. After 24 hours of incubation, the conditioned medium was collected, and the secreted levels of vascular endothelial growth factor (VEGF) and hepatocyte growth factor (HGF) were measured using the DuoSet ELISA Development System (R&D Systems, Minneapolis,MN). In addition, relative expression of *TGFβ1* was measured using the RNeasy mini kit (Qiagen, USA) to RNA extraction and complementary DNA was synthesized in a 20 μl reaction mixture using SuperScript III First-Strand Synthesis for RT-PCR (Invitrogen, USA). RT-qPCR was performed using SYBR Green Reagents (QPCR Master Mix, Agilent Technologies). All primer sets were previously screened for efficiency and their sequences were: *B2M* (F:5´ TCAGGTTTACTCACGTCATCC 3´, R:5' ACACGGCAGGCATACTCATC 3´), *TGFβ1* (F:5′ACAATTCCTGGCGATACCTCAGCA3′, F:5′TGCAGTGTGTTATCCCTGCTGTCA3′).

IDO activity

UC-MSCs and BM-MSCs cells were stimulated with 20 ng/mL of IFNγ and 10 ng/mL of IL-1β or with 100 ng/mL of IFNγ during 48 hours in Dulbecco's modified Eagle's medium (DMEM) supplemented with 2% fetal bovine serum, 2 mM L-glutamine, 1% penicillin/streptomycin (all reagents from Gibco, Gran Island, USA) and 100 ug/mL of L-tryptophan (Sigma-Aldrich, St Louis, MO,USA). IDO enzyme activity was measured determining the kynurenine content in the cell supernatant as previously reported[1,2].

IL6, TGF-β, PGE2, PDL-1 and HLA-G quantification

IL6, TGF-β and PGE2 were quantified in the supernatants of BM-MSCs and UC-MSCs stimulated for 48 hours in the absence and presence of 20 ng/mL IFN-γ and 10 ng/ml IL-1β in Dulbecco's modified Eagle's medium (DMEM) supplemented with 2% fetal bovine serum, 2 mM L-glutamine, 1% penicillin/streptomycin (all reagents from Gibco, Gran Island, USA), using an enzyme- linked immunosorbent assay (ELISA) from R&D Systems (R&D Systems, Minneapolis, MN,USA) following the manufacturer instructions. PDL-1 and HLA-G detection was performed using flow cytometry (FACSCanto™, BD Bioscience). BM-MSCs and UC-MSCs were collected and stained with specific antibodies (BD Pharmingen, San Jose, CA, USA) according to previously published staining procedures[3]. Briefly, The collected cells were resuspended in 100 μl of FACS buffer (PBS 1X, 0.2% BSA, 0.01% sodium azide) and incubated for 20 minutes at 4°C with the

appropriate fluorescently labeled monoclonal antibody directed against lymphocyte surface markers (BD Biosciences, CA, USA), washed and resuspended in FACS buffer, and analyzed by the FACS Canto II cytometer using the FACS Diva software (BD Biosciences, CA, USA). The viability was determined using LIVE/DEAD®Fixable dead cell stain kit (Invitrogen, CA, USA) according to the manufacturer's protocol. The data acquired was analyzed using the FlowJo software (Tree Star, Ashland, OR, USA).

T-Cell Subset Proliferation Assays

The proliferation of different T-cell subsets from 4 HFrEF patients included in this trial was performed in vitro to evaluate the immunomodulatory effect of UC-MSCs and BM-MSCs. Human peripheral blood mononuclear cells PBMCs were isolated by Ficoll-Paque Plus (GE Healthcare, Amersham, UK) (1.077 g/ml) density-gradient according to manufacturer's instruction. PBMCs were stained with carboxyfluorescein succinimidyl ester (CFSE; Life Technologies, Carlsbad, CA) following the manufacturer's protocol, and co-cultured with MSCs in 96-well plates at a 1:10 ratio (MSCs:hPBMC) in Roswell Park Memorial Institute (RPMI) medium (ThermoFisher, USA) supplemented with 10% FBS, 1% L-G, 1% nonessential amino acids (Sigma-Aldrich, USA), 100mM sodium pyruvate (Sigma, USA), 25 mMb-mercaptoethanol (Gibco, NY), and 15mg/ml phytohemagglutinin (PHA) (Sigma, USA). After 72 hours, PBMC were stimulated for 4 hours with 50 ng/ml phorbolmyristate acetate (PMA) (Sigma Aldrich) and 1µg/ml ionomycin (Sigma-Aldrich) in the presence of Brefeldin A (Biolegend, San Diego, Ca, USA). For surface staining, cells were incubated with antibodies against human CD4, CD8, CD3 and CD25 (BD Biosciences, USA) in the dark at 4°C for 30 min. Intracellular staining was performed using the BD Cytofix/Cytoperm solution, according to the manufacturer's protocol with antibodies against human IL-17, IL4 and IFNγ (eBioscience, USA). For transcriptional factor evaluation, we assessed FoxP3 expression with staining buffer and specific antibodies (eBioscience, USA) according to the manufacturer's protocol. Cells were acquired using a FACS Canto II Flow cytometer (BD Biosciences) and analyzed with the FlowJo software (Tristar, Stanford) for phenotype and proliferation, calculated by the decrease in CFSE fluorescence.

Migration capacity

Cell migration assays were performed with the Transwell two-chamber cell culture method (Corning, Cambridge, MA) with an 8 µm pore polycarbonate membrane. The uppermost side of the Transwell membrane was coated with 0.1% gelatin in PBS (Sigma-Aldrich, St. Louis, MO) for 2 h at 37℃. UC-MSCs or BM-MSCs were seeded at a density of 15.000 cells per 100 µl of DMEM 1% P/S, 0.1%FBS in the upper chamber of the Transwell apparatus. Cells were allowed to migrate toward medium (500 µl) in the lower chamber containing DMEM alone or supplemented with 5% of serum isolated from the HFrEF patients. The Transwell system was incubated for 16h at 37℃ in a humidified atmosphere containing 5% CO2. After incubation, non-migratory cells were carefully removed from upper face of the Transwell insert with a cotton swab. The attached cells remaining on the Transwell insert were fixed with 70% methanol and stained with 1% crystal violet in 20% methanol for 1 h. After washing, the stained cells that migrated from the upper to the lower side of the membrane were counted under an inverted bright-field microscope at 20X magnification. The number of migrated cells was expressed as the percent change from the control value (DMEM alone). Each experiment was performed in biological and experimental triplicate.

SUPPLEMENTAL RESULTS

Cellular size

Parameters defined for the MSC setting of the Countess™ (Invitrogen) automated cell counter included control of circularity and maximum-minimum cell size (applied gate 10um - 28um). The analysis of 26 therapy releases resulted in an average size measurement of UC-MSC´s of 17.1 ± 3.5 μm.

Doubling time

The doubling time (DT) data were collected from each of the cell culture passage performed under GMP conditions. The assessment of 50 samples resulted in an average DT of 32.6 ± 8.8 hours.

Senescence-associated beta-galactosidase assay

UC-MSC and BM-MSC senescence according to the expression of SA-β-galactosidase was evaluated under standard culture conditions normalized for passage and culture duration. UC-MSC showed around 2 fold less senescent cells than BM-MSC´s (Online Figure II).

Quantification of IDO activity, IL6, TGF-β1, PGE2 and PDL-1 at basal and stimulated condition

The expression of these mediators involved in the regenerative and suppressive effects of MSCs was evaluated for both cell sources (BM or UC-MSCs) in baseline conditions and after proinflammatory stimulus with IFNγ and IL-1β at optimal conditions (20 and 10 ng/ml respectively). UC-MSCs expressed similar levels of PDL-1, HLA-G as well as IDO activity compared to BM-MSCs under the different culture conditions. Furthermore, both MSC sources responded to the IFNγ + IL-1β stimulated conditions by an increased protein expression levels of IL-6, TGF-β1 and PGE2 (Online Figure III). Of interest, UC-MSCs expressed higher constitutive levels of PGE2 and TGF-β1, a molecule known for the induction of T regulatory (Treg) CD4+ cells and inhibition of NK function.

ONLINE FIGURE LEGENDS

Online Figure I. UC- MSCs expressed all the common MSC markers and demonstrate capacity to differentiate into chondrogenic, osteogenic and adipogenic lineage. (A): UC-MSCs were stained with labeled monoclonal antibodies against known MSC surface markers (blue) and their respective isotypes (red); the cells were analyzed by flow cytometry. All UC-MSCs were positive for CD105, CD73, CD90, CD44, CD146, CD49a and HLA-ABC but negative for CD14, CD34, CD31, CD45, and HLADR. (B) Representative images of UC-MSC differentiation after specific inductions and staining: adipocytes (Oil Red O), osteocytes (alizarin red), and chondrocytes (safranin O). Scale bars = 200 mm. All data are presented as mean ± SEM (n=3) of a minimum of 4 donors. Abbreviations: MSCs, mesenchymal stem cells; UC, umbilical cord.

Online Figure II. Size, doubling time and senescence of UC-MSCs. A. Cell preparations were counted using an automated cell counter assessing viability, cell counting and size measurements using the trypan blue method of dead-cell staining combined with advanced image analysis. The parameters for MSC setting included setting of the circularity and maximum and minimum cell size (applied gate 10um - 28um). The analysis of 26 therapy releases resulted in the average size measurement of 17.1±3.5 μM. B. The doubling time (DT) of UC-MSC. The data were collected from each cell culture process performed under GMP conditions. Doubling time was calculated based on initial cell number, culture time (hours) and final number of cells harvested. The assessment of 50 samples resulted in the doubling time of 32.6± 8.8 hours. C. Senescence-associated beta-galactosidase. UC-MSC and BM-MSC (n=3) were cultured and harvested under standard conditions. Positive stained cells for SA-β-galactosidase were counted and the percentage of cells expressing SA-β-galactosidase calculated over the total cell number of each sample analyzed. Under normalized culture condition (number of passages, and duration of culture), UC-MSC showed around 2 folds less senescent cells.

Online Figure III. BM and UC-MSCs immunoregulatory response to cytokine activation and expression of immunosuppressive molecules. A) IDO activity measured by kynurenine production. B) PDL-1 and HLA-G expression levels determined by flow cytometry (left), representative cytometry plots (right). C) PGE2, TGFβ1 and IL6 expression levels measured by ELISA. IDO activity were quantified in the supernatants of MSCs cultured in the absence (control group) or presence of 20 ng/ml IFNγ and 10ng/ml IL1β or 100 ng/ml of IFNγ. PDL-1, PGE2, TGF-β1 and IL6 expression levels were quantified on MSCs cultured in the absence (control group) or presence of 20 ng/ml IFNγ and 10ng/ml IL1β. Results are represented as mean±SEM of three independent experiments using each time three different UC-MSCs and BM-MSC donors. *, p<.05; **, p<.01; ***, p<.005 compared to MSCs control group without proinflammatory cytokines treatment. #, p<.05; ##, p<.01, UC-MSCs compared to BM-MSCs. Abbreviations: UC-MSCs, umbilical cord-derived mesenchymal stem cells; BM-MSCs, bone marrow-derived mesenchymal stem cells; IL, interleukin; IFN, interferon; IDO, indoleamine 2,3 dioxygenase; PDL-1, programmed cell death-ligand; HLA-G, histocompatibility antigen, class I, G; MFI, mean fluorescence intensity.

Online TABLES

Online Table I. Electrophysiological assessment of arrhythmogenic potential.

Variable	Group	N	Baseline	3 months	6 months	12 months
**QTC**	**Placebo**	14	453.9±32.1	442.1±30.6	446.6±36.4	449.4±29.7
	**UC-MSC**	14	437.5±43.5	449.8±27.3	449.5±36.3	440.9±30.0
**Lown class**	**Placebo**	14	2.57±1.50	2.36±1.50	1.85±1.46	2.14±1.46
	**UC-MSC**	14	1.43±1.16*	1.71±1.27	1.57±1.22	1.50±1.09
**PVC**	**Placebo**	14	550±1090	904±1535‡	1526±3657	1875±3052†
	**UC-MSC**	14	385±970	394±669	1294±1955	967±2100
**LP §**	**Placebo**	9	0.67±0.86	0.78±0.83	0.22±0.67	0.44±0.88
	**UC-MSC**	10	1.20±1.13	0.70±0.95	0.80±0.92	0.30±0.67†
**SDNN**	**Placebo**	14	35.35±17.53	36.96±20.21	39.43±31.19	43.94±27.89
	**UC-MSC**	14	52.89±52.06	42.29±47.91‡	49.43±60.84	43.25±49.52
**RMSSD**	**Placebo**	14	28.10±21.50	34.06±23.48	30.21±20.13	34.06±24.03
	**UC-MSC**	14	62.85±99.90	43.89±71.04	58.58±88.72	65.70±97.69
**HRVTI**	**Placebo**	14	11.76±4.86	9.54±3.82†	12.12±6.78	11.34±5.18
	**UC-MSC**	14	11.97±6.53	11.04±7.08	10.77±8.92	20.45±36.22
**LF**	**Placebo**	14	103.3±47.6	89.51±41.17	97.26±78.28	95.40±54.58
	**UC-MSC**	14	111.2±34.3	111.0±46.6	108.65±64.08	99.12±36.51
**HF**	**Placebo**	14	104.7±52.5	126.0±80.6	95.6±60.3	109.2±78.7
	**UC-MSC**	14	160.9±88.4	138.7±87.1	167.9±103.2	149.1±99.6
**LF/HF ratio**	**Placebo**	14	1.32±1.12	2.04±3.75	2.09±3.77	1.32±1.16
	**UC-MSC**	14	1.24±1.52	1.51±1.86	1.03±0.82	1.04±0.77

QTC: Corrected QT segment according to Bazett's formula. PVC: Total number of premature ventricular contractions in 24-hour ECG Holter test. LP: Number of late potentials criteria according to signal averaged ECG. SDNN: Standard deviation of NN intervals. RMSSD: Root mean square of successive differences. HRVTI: Heart Rate Variability Triangular Index. LF: Low Frequency ranges. HF: High Frequency ranges. *p<0.05 versus placebo. †p<0.05 versus baseline. ‡p<0.0167 versus baseline. § Patients with bundle branch block or atrial fibrillation were excluded from this analysis.

Online Table II. Additional parameters of exercise capacity assessed at cardiopulmonary test.

Variable	Group	N	Baseline	3 months	6 months	12 months
**METS**	**Placebo**	14	5.01±1.43	4.76±1.47	5.10±1.40	5.35±1.46
	**UC-MSC**	14	5.17±1.34	5.40±1.13	5.31±1.38	5.11±1.18
**VT**	**Placebo**	14	12.42±3.29	12.44±4.00	12.45±2.88	13.52±3.02
	**UC-MSC**	14	12.32±3.43	13.60±5.36	13.30±5.99	13.52±5.49
**RER**	**Placebo**	14	1.22±0.20	1.13±0.09†	1.16±0.09	1.13±0.10
	**UC-MSC**	14	1.13±0.10	1,13±0.09	1.09±0.08	1.09±0.09
**Exercise time (min)**	**Placebo**	14	6:41±1:24	6:17±1:52	6:18±1:35	7:03±2:11
	**UC-MSC**	14	6:51±2:40	6:35±2:05	6:48±2:23	6:46±2:15

VT: VO2 at anaerobic threshold (ml/Kg/min). RER; peak respiratory exchange ratio. *p<0.05 versus placebo. †p<0.05 versus baseline.

ONLINE SUPPLEMENT REFERENCES

1. Däubener W, Hucke C, Seidel K, Hadding U, MacKenzie CR. Interleukin-1 inhibits gamma interferon-induced bacteriostasis in human uroepithelial cells. *Infect Immun*. 1999;67:5615–20.
2. Luz-Crawford P, Torres MJ, Noël D, Fernandez A, Toupet K, Alcayaga-Miranda F, Tejedor G, Jorgensen C, Illanes SE, Figueroa FE, Djouad F, Khoury M. The immunosuppressive signature of menstrual blood mesenchymal stem cells entails opposite effects on experimental arthritis and graft versus host diseases. *Stem Cells*. 2016;34:456–69.
3. Alcayaga-Miranda F, Cuenca J, Martin A, Contreras L, Figueroa FE, Khoury M. Combination therapy of menstrual derived mesenchymal stem cells and antibiotics ameliorates survival in sepsis. *Stem Cell Res Ther*. 2015;6:199.

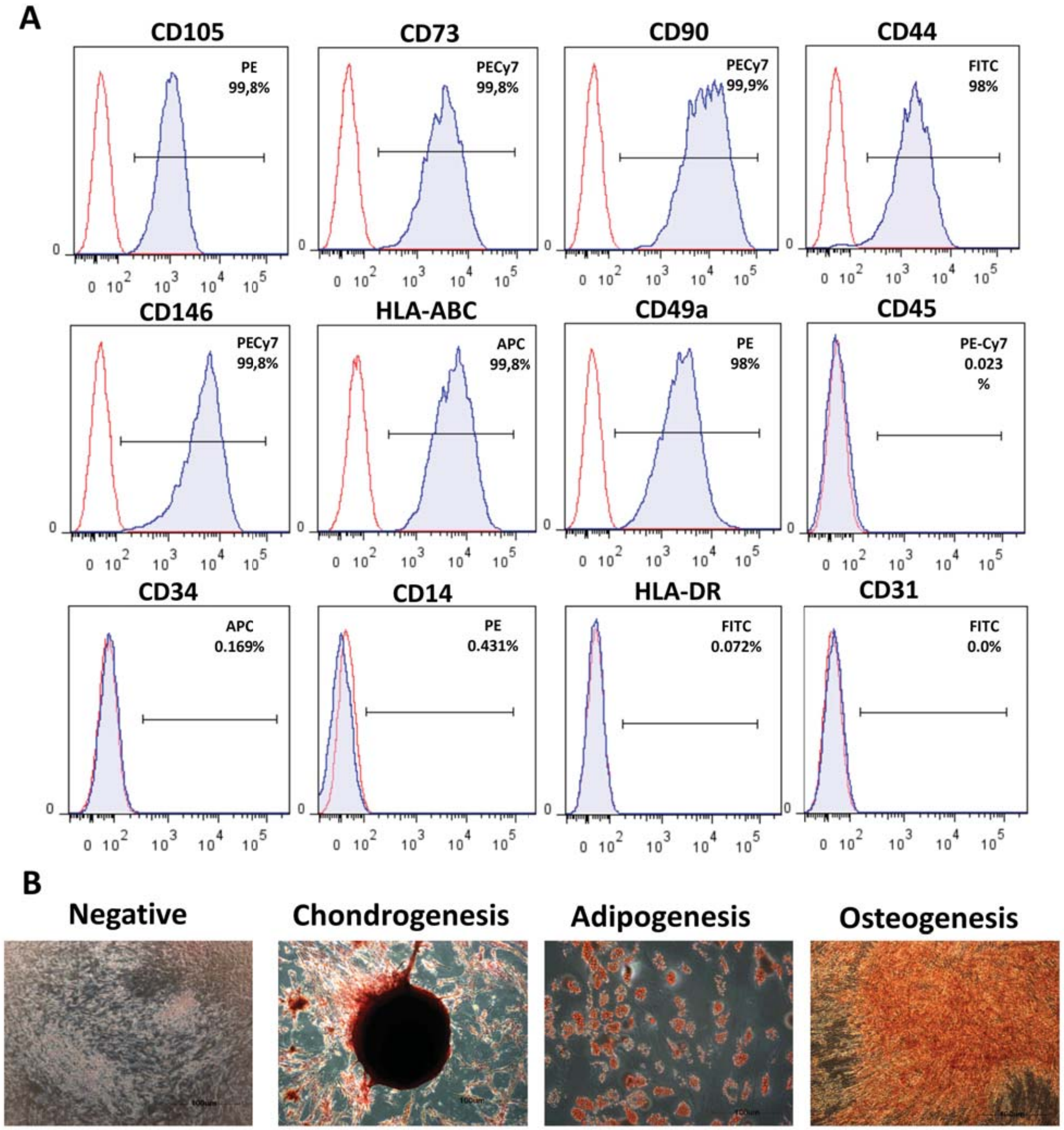
A
CD105
PE
99,8%
CD73
PECy7
99,8%
CD90
PECy7
99,9%
CD44
FITC
98%
CD146
PECy7
99,8%
HLA-ABC
APC
99,8%
CD49a
PE
98%
CD45
PE-Cy7
0.023
%
CD34
APC
0.169%
CD14
PE
0.431%
HLA-DR
FITC
0.072%
CD31
FITC
0.0%
B
Negative
Chondrogenesis
Adipogenesis
Osteogenesis

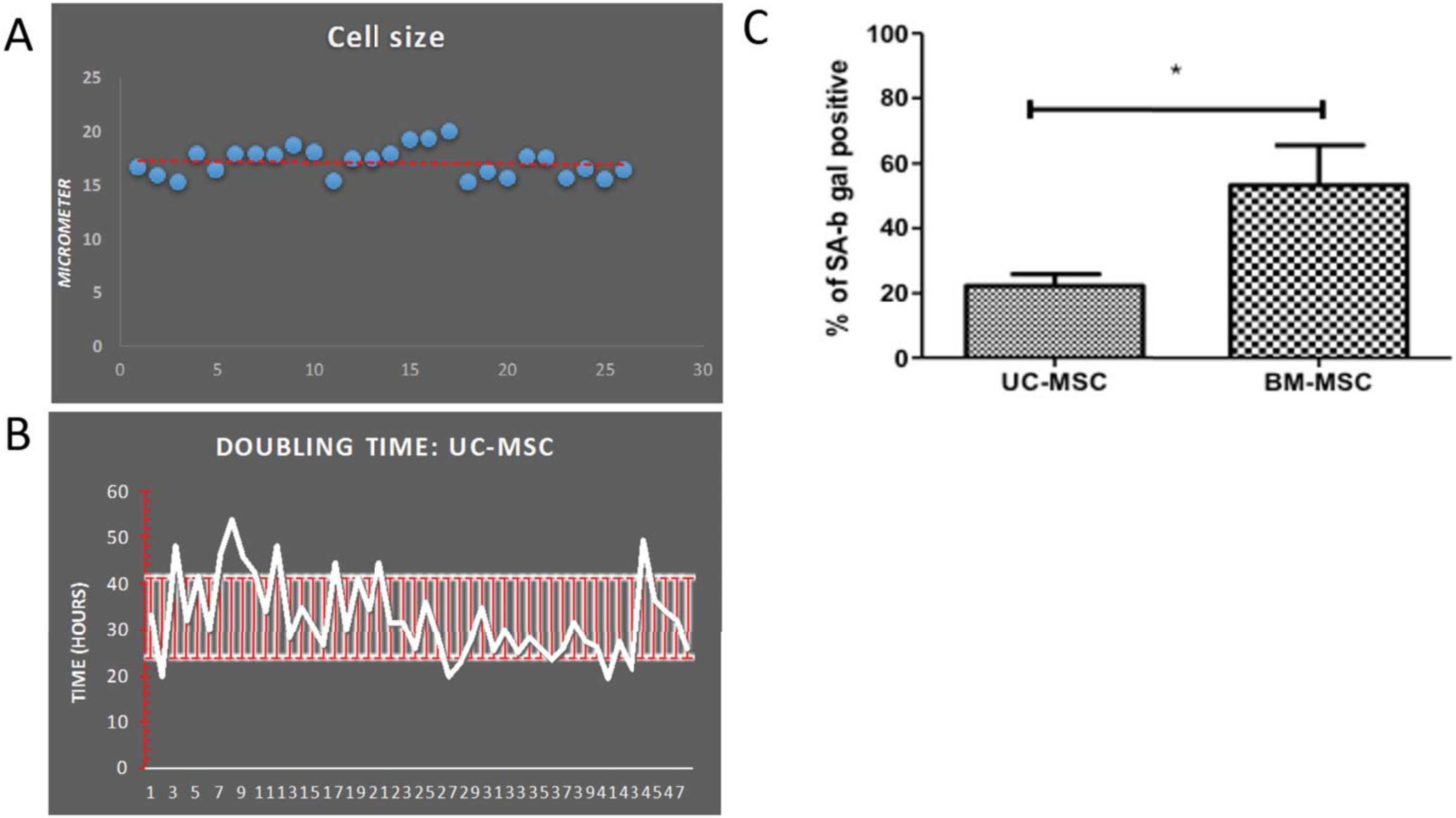
A
Cell size
MICROMETER
B
DOUBLING TIME: UC-MSC
TIME (HOURS)
C
% of SA-b gal positive
*
UC-MSC
BM-MSC

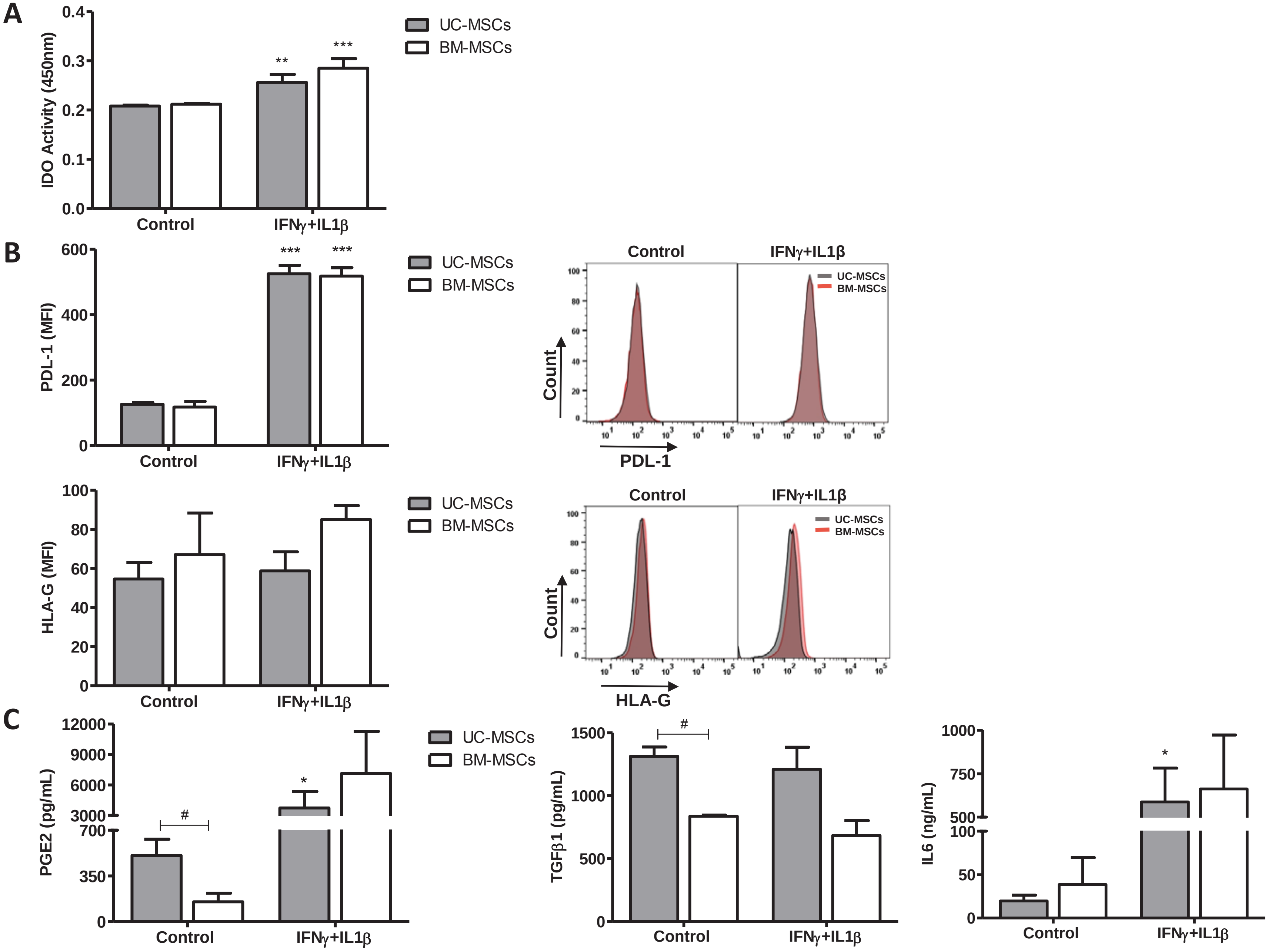
A
IDO Activity (450nm)
0.0
0.1
0.2
0.3
0.4
**
***
Control
IFNγ+IL1β
UC-MSCs
BM-MSCs
B
PDL-1 (MFI)
0
200
400
600
***
***
Control
IFNγ+IL1β
UC-MSCs
BM-MSCs
Control
IFNγ+IL1β
UC-MSCs
BM-MSCs
Count
PDL-1
HLA-G (MFI)
0
20
40
60
80
100
Control
IFNγ+IL1β
UC-MSCs
BM-MSCs
Control
IFNγ+IL1β
UC-MSCs
BM-MSCs
Count
HLA-G
C
PGE2 (pg/mL)
0
350
700
3000
6000
9000
12000
#
*
Control
IFNγ+IL1β
UC-MSCs
BM-MSCs
TGFβ1 (pg/mL)
0
500
1000
1500
#
Control
IFNγ+IL1β
IL6 (ng/mL)
0
50
100
500
750
1000
*
Control
IFNγ+IL1β
